# Supplementary material for: EMDA: A Python package for Electron Microscopy Data Analysis
Source: J Struct Biol. 2022 Mar;214(1):107826. doi: 10.1016/j.jsb.2021.107826 (PMC8935390; doi:10.1016/j.jsb.2021.107826)
Supplement: Supplementary Data 1 [file mmc1.docx]

Supplementary materials

**EMDA: A Python package for Electron Microscopy Data Analysis**

**Rangana Warshamanage^*^, Keitaro Yamashita & Garib N. Murshudov^*^**

Structural Studies, MRC Laboratory of Molecular Biology, Francis Crick Avenue, Cambridge CB2 0QH, England

* To whom correspondence may be addressed: ranganaw@mrc-lmb.cam.ac.uk, garib@mrc-lmb.cam.ac.uk

**Relationship between scaling and blurring**

Let us assume that there are two maps - $\psi_{1}(x)$ and $\psi_{2}(x)$ with corresponding Fourier coefficients - $F_{1}(s)$ and $F_{2}(s)$. Usually, to compare these maps or to calculate differences between them these maps are scaled, i.e. one of the Fourier coefficients is multiplied with resolution dependent scale factor, $k(s)$. I.e. $F_{1}\left( s \right)$ and $k\left( s \right) F_{2}(s)$ and/or their inverse transformations compared. According to the convolution theorem multiplying Fourier coefficients with a resolution dependent scale factor is equivalent to averaging the density at each point using the kernel derived from the inverse Fourier transformation of the scale factor:

$$\mathcal{F}^{-1}\left( k\left( s \right)F_{2}\left( s \right) \right)= \int_{y\in R^{3}} \psi_{2}\left( y \right)K\left( x-y \right)d^{3}y$$

Where $K\left( x \right)=\mathcal{F}^{-1}\left( k\left( s \right) \right)$. For this reason, often deblurring and scaling are used interchangeably. Thus, scaling in Fourier space is the same as blurring/sharpening in real space. If the scale factor is Gaussian then the blurring/sharpening kernel is also Gaussian.

In practice, if there are more than one maps they can be scaled to each other.

**Kernel with different radii used in local correlation examples**

Note that these are central slices of 3D kernels before normalisation.


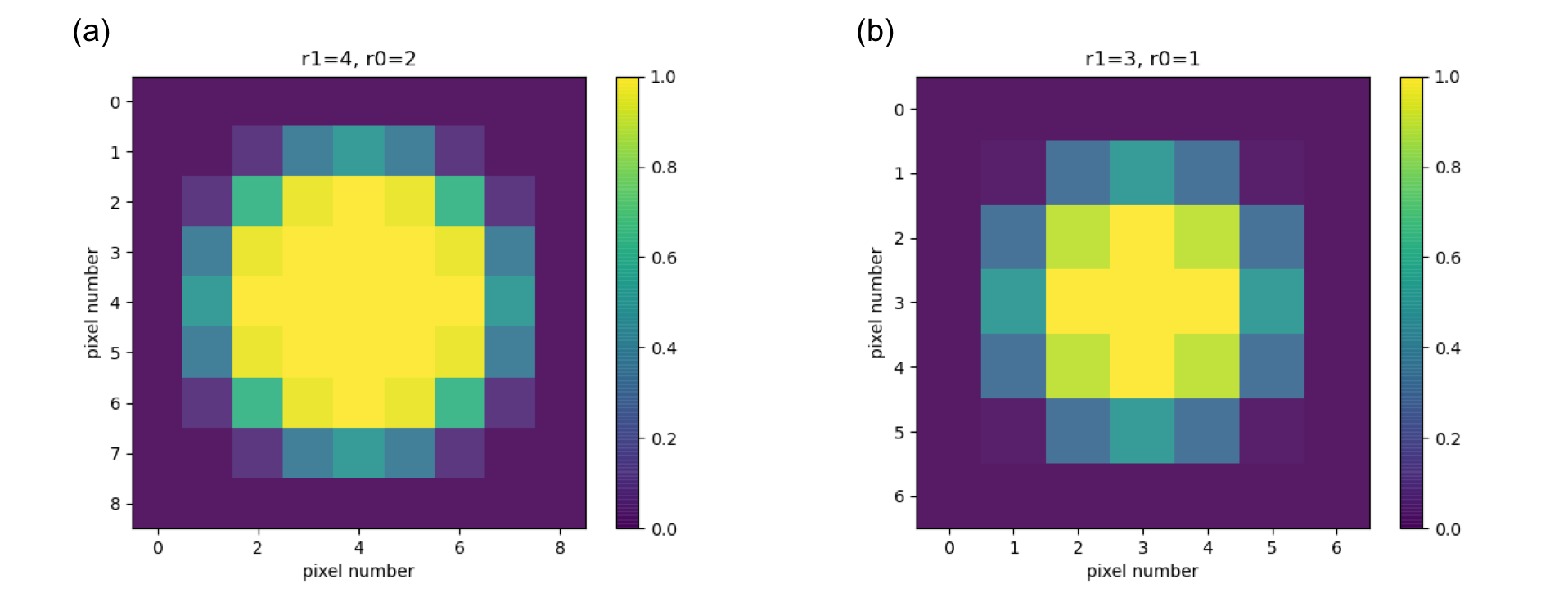


Fig. S1. 2-dimensional projections of 3D kernels used in correlation examples. a) kernel used in example 1, b) used in example 2.

**Effect of kernel size in local correlation**

Fig. S2. Effect of kernel size in local correlation example 1.

Fig. S3. Effect of kernel size in local correlation example 2
